# Biomechanical Design and Validation of a Novel Elliptical Sleeve Pedicle Screw for Enhanced Spinal Fixation Stability

**DOI:** 10.3390/bioengineering12060668

**Published:** 2025-06-18

**Authors:** Ting-Shuo Hsu, Chang-Jung Chiang, Hsuan-Wen Wang, Yu-San Chen, Chun-Li Lin

**Affiliations:** 1 Department of Biomedical Engineering, National Yang Ming Chiao Tung University, Taipei 112304, Taiwan; b101093048@tmu.edu.tw (T.-S.H.); 411918@gmail.com (H.-W.W.); xw9672gi24@gmail.com (Y.-S.C.); 2 Department of Orthopaedics, Shuang Ho Hospital, Taipei Medical University, New Taipei City 235041, Taiwan; cjchiang@s.tmu.edu.tw; 3 Department of Orthopaedics, School of Medicine, College of Medicine, Taipei Medical University, Taipei 110301, Taiwan; 4 Innovation & Translation Center of Medical Device, Department of Biomedical Engineering, National Yang Ming Chiao Tung University, Taipei 112304, Taiwan

**Keywords:** pedicle screw, ellipse, pull-out, biomechanics, fatigue

## Abstract

This study aimed to develop a novel modular pedicle screw system incorporating an elliptical sleeve to conform the pedicle’s elliptical cross-section and enhance fixation strength with mechanical stability. The biomechanical evaluation was conducted based on fundamental mechanics principles, followed by a finite element (FE) analysis to assess stress distribution under compressive and torsional loads. Subsequently, mechanical testing was performed to evaluate static and fatigue bending performance and in vitro biomechanical fatigue in porcine vertebrae by pull-out testing after 5000 and 100,000 cycles to assess fixation stability. The FE analysis demonstrated that the elliptical sleeve design improved bending resistance by 1.21× and torsional resistance by 1.91× compared to conventional cylindrical screws. Mechanical testing revealed greater bending/torsion stiffness and fatigue resistance, with the elliptical sleeve screw withstanding 5 million cycles at 235.4 N, compared to 175.46 N for cylindrical screws. Biomechanical pull-out testing further confirmed significantly higher retention strength after 100,000 cycles (1229.75 N vs. 867.83 N, *p* = 0.0101), whereas cylindrical screws failed prematurely at 10,663 cycles due to excessive displacement (>2 mm). The elliptical sleeve pedicle screw system demonstrated enhanced fixation strength, reduced micromotion, and superior fatigue resistance, making it a promising alternative to conventional pedicle screws for improving long-term spinal fixation stability.

## 1. Introduction

Pedicle screws are among the most commonly used internal fixation devices in spinal surgery, providing stability by anchoring securely to the pedicle and vertebral body [1]. They play a critical role in treating degenerative spinal diseases, trauma, tumors, deformities (e.g., scoliosis), and infections [1,2,3,4]. Their primary function is to reduce intervertebral motion, promote bone fusion, and restore spinal function. In addition, pedicle screw systems are often integrated with rods or cross-link devices to form stable constructs that meet the demands of complex spinal disorders. Despite their widespread application, clinical failure rates of pedicle screws range between 11–17% [5,6,7,8], with common failure modes including loosening, breakage, pull-out, and cut-out [9,10,11,12]. These failures are often linked to biomechanical factors such as inadequate bone–screw interface strength, particularly in cases of thin cortical bone or low bone density (e.g., osteoporosis), which compromises fixation stability [13].

**Figure 1:**
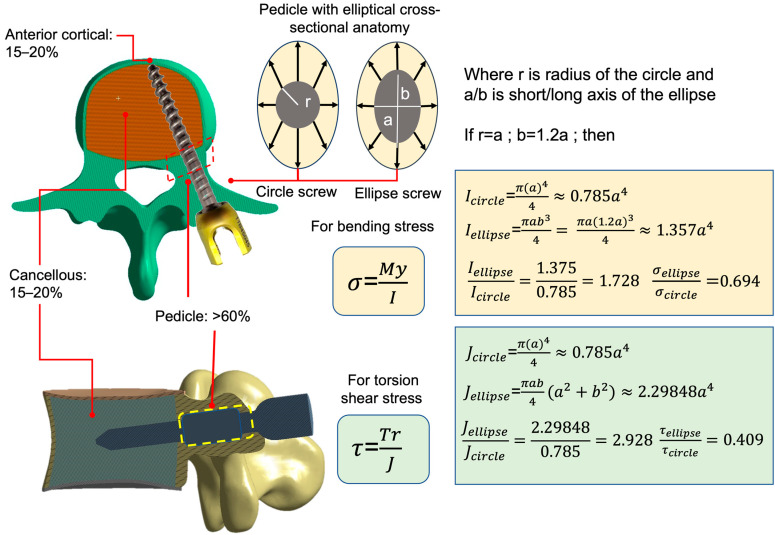
(**Left**): The distribution of force across different regions after pedicle screw implantation in the spine. (**Right**): Bending stress and torsional shear force calculation formulas, along with the impact ratio of the second moment of inertia between elliptical and cylindrical screws.

The mechanical contribution of different spinal regions to pedicle screw fixation has been experimentally studied. Weinstein et al. [14] reported that in resisting axial tensile strength, the pedicle contributes approximately 60% of the fixation strength, while the vertebral body accounts for 15–20%, and the anterior cortical bone (bicortical fixation) contributes another 15–20% (Figure 1). Similarly, Hirano et al. [15] found that the pedicle provides 80% of the rigidity in resisting spinal “flexion-extension” forces and 60% of the strength against pedicle screw pull-out compared to the vertebral body (Figure 1). Furthermore, Uvaraj et al. [16] demonstrated that stacking screws in the pedicle region, following the anatomical shape of the pedicle, can offer effective repair after screw failure. These findings highlight that the biomechanical performance of screws at the pedicle is a critical factor in determining the success or failure of pedicle screws. The pedicle’s structural integrity and its ability to anchor screws effectively play a decisive role in ensuring long-term stability and preventing failure.

From an anatomical perspective, the pedicle’s elliptical cross-sectional anatomy presents opportunities to improve screw design. Material mechanics suggest that screws with elliptical cross-sections could provide a higher second moment of inertia, enabling greater stress resistance and more even force distribution to surrounding bone (Figure 1) [17]. This design could reduce the risk of screw loosening, displacement, or fracture. However, current commercially available screws feature cylindrical cross-sections, with design innovations primarily focusing on thread patterns, expansion capabilities, and cement augmentation, neglecting the significance of the pedicle’s elliptical structure [18,19,20,21,22]. Furthermore, while elliptical screws could enhance stability, their asymmetric shape presents implantation challenges, as traditional screw-in methods may not apply. Press-fit techniques, while feasible, may compromise the bone–screw interface strength.

This study proposes a new design concept for pedicle screws, aiming to improve upon the traditional cylindrical shape by incorporating an elliptical sleeve aligned with the pedicle’s anatomy. Using specifically designed bone-shaping instruments, the modular pedicle screw system can be effectively secured within the pedicle and vertebral body, achieving superior mechanical performance. Finite element (FE) analysis was conducted to confirm that the elliptical structure at the pedicle outperforms the cylinder design in terms of mechanical properties. Subsequently, a new pedicle screw/elliptical sleeve combination and corresponding implantation tools were manufactured and subjected to FDA-compliant static compression/torsion and compression fatigue tests. Finally, in vitro biomechanical fatigue compression and pull-out tests were conducted using porcine spines implanted to validate the feasibility of this novel design.

## 2. Materials and Methods

### 2.1. FE Analysis of Cylinder and Ellipse Pedicle Screws

**Figure 2:**
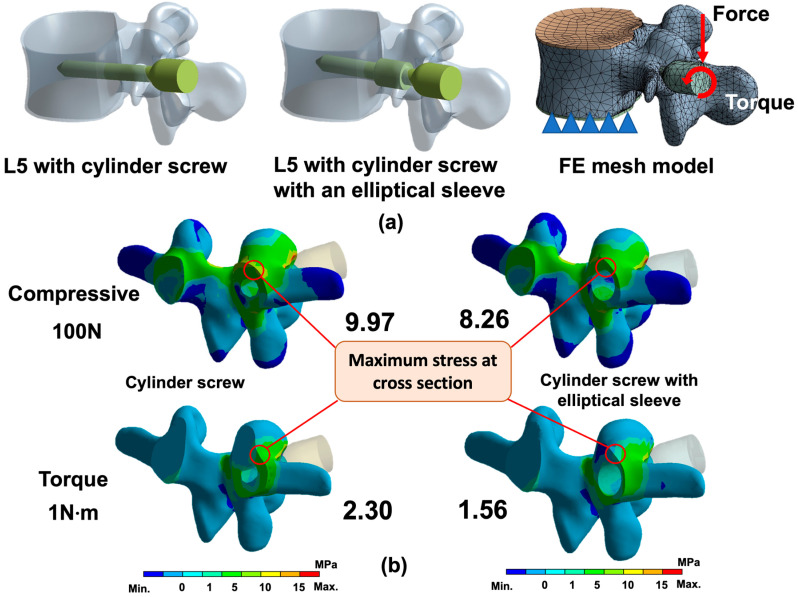
(**a**) (**Left**): Solid model of the implanted cylindrical screw; (**middle**): solid model of the implanted elliptical screw; (**right**): FE model with applied forces and boundary conditions. (**b**) FE analysis results, showing the maximum principal stress distribution at the pedicle cross-section under compressive and torsional loads for both cylindrical and elliptical screws.

From a previously constructed 3D model of the lumbar spine (L2 to L5), the L5 segment, including cortical bone, cancellous bone, and endplates, was extracted and reconstructed in CAD software (Creo Parametric v2.0, PTC, Needham, MA, USA) [23]. On the left side, cylinder pedicle screw and with an elliptical sleeve at pedicle were modeled and implanted through the pedicle into the vertebral body. The cylindrical screw had a diameter of 4.5 mm, a length of 35 mm, and its threads were simplified to a cylindrical shape. The elliptical pedicle screw was based on the cylindrical screw but featured an elliptical addition at the pedicle region. This elliptical cross-section had a major axis of 5.4 mm (in conjunction with the 4.5 mm pedicle screw, it was 1.2 times the size of the 4.5 mm) and a minor axis of 5 mm, extending 10 mm in length (Figure 2a). The elliptical addition was designed to pass through the narrowest part of the pedicle and simulate implantation with an elliptical cross-section at the pedicle.

These two FE models were created using quadratic ten-node tetrahedral structural solid elements, comprising 12,086 elements and 24,721 nodes for the cylindrical screw model, and 25,250 elements and 47,063 nodes for the elliptical screw model, in the FE package (ANSYS Workbench v18.2, ANSYS Inc., Canonsburg, PA, USA) for simulation (Figure 2a). The cortical bone, cancellous bone, endplates, and titanium alloy screws were assigned linear elastic and isotropic material properties based on relevant literature [23] (Table 1). Nodes on the bottom of the L5 endplate were constrained in all directions as boundary conditions. A vertical compressive force of 100 N and a torque of 10 N·m were applied at the end of both pedicle screws to evaluate the maximum bone stress (1st principal stress) at the cross-section of the pedicle (Figure 2a).

### 2.2. Design and Manufacture of Novel Pedicle Screw System

**Figure 3:**
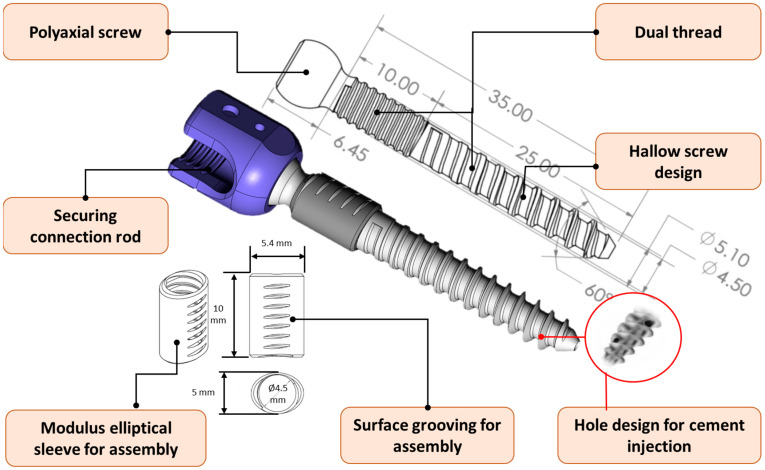
New elliptical pedicle screw, including a cylindrical body and an elliptical sleeve design engineering drawing.

The new pedicle screw system was designed to retain the advantages of traditional cylindrical screws while incorporating the biomechanical benefits of an elliptical sleeve, aiming to enhance fixation strength at the pedicle. The system adopted an innovative modular design, consisting of a screw body and a modular elliptical sleeve. The screw body featured a poly-axial design at the screw head, enabling multi-axial adjustment to adapt flexibly to various spinal anatomies and securely connect to the rod, ensuring a stable connection with fixation devices. The screw body had a total length of 35 mm and utilized a dual thread design, with fine threads in the cortical bone contact region (approximately 10 mm in length and 5.1 mm in diameter, pitch 1.2 mm) and coarse threads in the cancellous bone contact region (approximately 25 mm in length and 4.5 mm in diameter, pitch 2.4 mm) to improve bone anchorage. Additionally, the screw incorporated a hollow design (hole with 1.5 mm in diameter), which provided a channel for bone cement injection to address challenges associated with osteoporosis or low bone density (Figure 3).

The modular elliptical sleeve was specifically tailored to match the anatomical elliptical cross-section of the pedicle. This design effectively increased the contact area between the screw and the surrounding bone, evenly distributed stress, and reduced the risks of screw loosening or displacement. The detailed dimensions of the elliptical cross-section are shown in Figure 3. The inner structure of the sleeve consists of a hollow cylinder designed to interlock with a 4.5 mm pedicle screw, while the outer structure features a 5.0 mm × 5.4 mm elliptical profile with a length of 10 mm, intended to achieve a snug fit within the pedicle. Its surface incorporated several grooves, which enabled secure integration with the fine threads of the screw body during insertion, forming a stable and efficient assembly structure (Figure 3). The novel elliptical sleeve screw design—including its shape, dimensions, and the corresponding surgical instrument—has been submitted to the patent office for intellectual property protection (Taiwan patent I813158).

**Figure 4:**
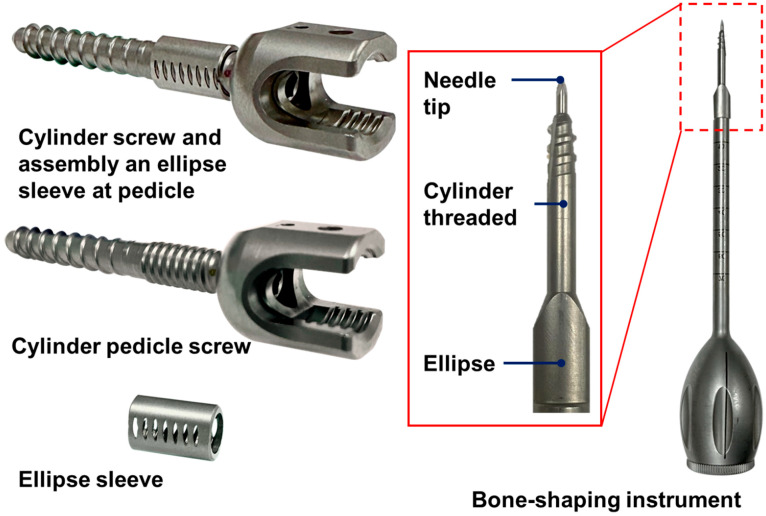
(**Left**): Completed fabrication of the new elliptical screw system, including the cylindrical body and elliptical sleeve. (**Right**): Completed fabrication of the customized bone-shaping instrument.

After the design of the pedicle screw system was completed, manufacturing was outsourced to a professional company equipped with a Quality Management System (QMS) to ensure product stability and consistency. The manufacturing process involved high-precision CNC lathing to machine the cylindrical body of the screw, as well as the fine and coarse threads, while a drill was used to create a hollow structure for bone cement infusion. The screw head was processed using a five-axis machining center to achieve the poly-axial structure and hexagonal socket. The elliptical sleeve was precisely machined with a CNC milling machine to form the elliptical cross-section and surface grooves, ensuring a secure fit with the screw threads. The finished screws and sleeves underwent deburring, anodizing, and polishing treatments, resulting in high-quality products that meet medical device standards (Figure 4).

### 2.3. Design of the Specific Instrument & Implantation Procedure

**Figure 5:**
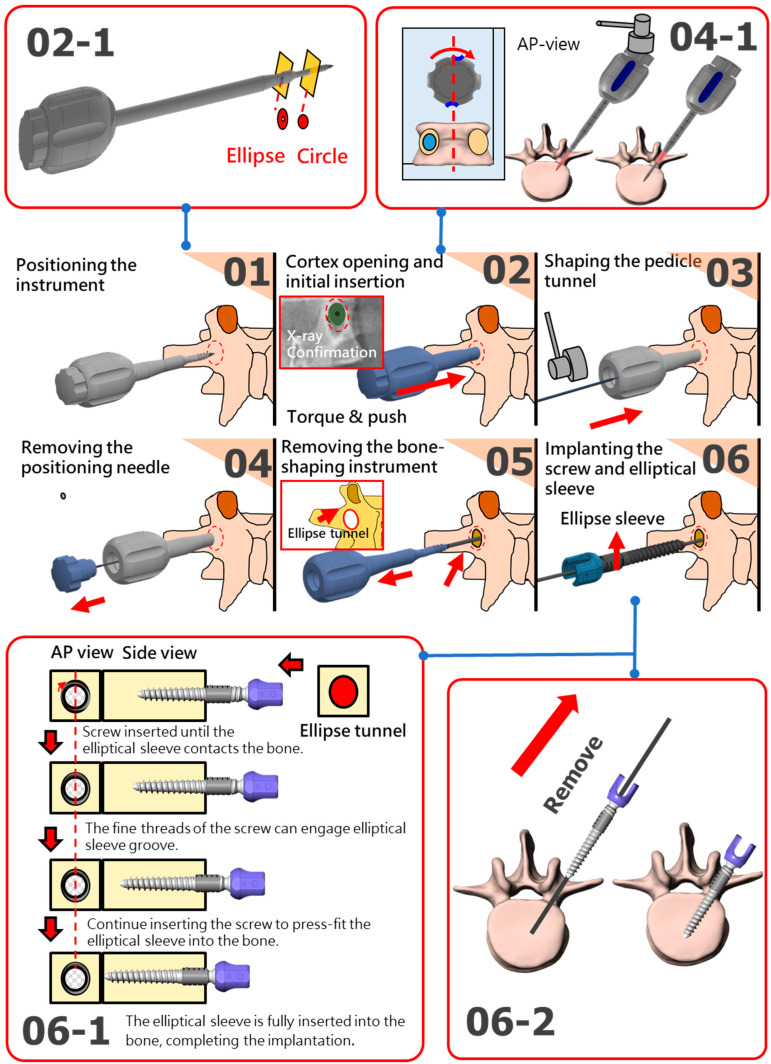
Illustrations of the implantation process for the pedicle screw system involved six key steps.

To effectively implant the elliptical sleeve of the new pedicle screw system into the pedicle, a specially designed bone-shaping instrument was required to create an elliptical tunnel. This instrument featured a cylindrical threaded design and an elliptical body at the front, a handle at the rear, and an integrated positioning needle (Figure 5). The implantation process for the pedicle screw system involved six key steps:

1. Positioning the instrument: Intraoperative imaging (e.g., X-ray or C-arm) was used to align the tip of the bone-shaping instrument with the pedicle entry point, and a small hole was created to mark the insertion site (Step 1 in Figure 5).
2. Cortex opening and initial insertion: After confirming the entry point, the front end of the bone-shaping instrument was used to open the surface cortex. The instrument was then inserted into and through the isthmus of the pedicle with appropriate torque and pressure. The medio-lateral projection angles should be adjusted according to the trajectory of the pedicle to avoid the tip of the bone-shaping instrument penetrating beyond the medial wall of the pedicle cortex. At this stage, the elliptical portion of the instrument remained outside the pedicle (Step 2 in Figure 5).
3. Shaping the pedicle tunnel: A mallet was used to drive the bone-shaping instrument further into the pedicle, sculpting the bone into an elliptical tunnel. Alignment lines on the instrument ensured that the long axis of the elliptical tunnel was parallel to the long axis of the pedicle. (Step 03 in Figure 5).
4. Removing the positioning needle: Once the shaping was complete, the internal positioning needle was removed from the pedicle channel. A guide pin was inserted into the tunnel to guide the subsequent screw implantation. (Step 04 in Figure 5).
5. Removing the bone-shaping instrument: The bone-shaping instrument was removed, leaving an elliptical cross-section in the pedicle. This tunnel was now ready for the integration of the elliptical sleeve and screw (Step 05 in Figure 5).
6. Implanting the screw and elliptical sleeve: The pedicle screw, with its coarse-threaded portion, was screwed in until the elliptical sleeve contacted the pedicle. The sleeve was then press-fitted into the elliptical cavity without rotation, ensuring a parallel fit. Simultaneously, the fine-threaded portion of the screw engaged with the grooves of the elliptical sleeve. The screw was advanced further, fully seating the sleeve into the pedicle until it reached the intended position. Finally, the guide pin was removed, completing the implantation process (Step 06, 06-1, and 06-2 in Figure 5).

The aforementioned specific bone-shaping instrument was also manufactured by a QMS-certified company to ensure compliance with the high-quality requirements for medical devices (Figure 4).

### 2.4. ASTM F1717 Testing for Static/Fatigue Compression Bending and Static Torsion

Pedicle screws are typically combined with rods and other components to form a complete spinal implant assembly, which provides stability during the arthrodesis process. ASTM F1717 is the FDA-recommended standard test method for evaluating and quantifying the mechanical performance of different designs of spinal implant assemblies [24]. According to FDA recommendations, lumbar pedicle screw systems are required to undergo axial compressive bending static/fatigue tests and static torsion tests.

**Figure 6:**
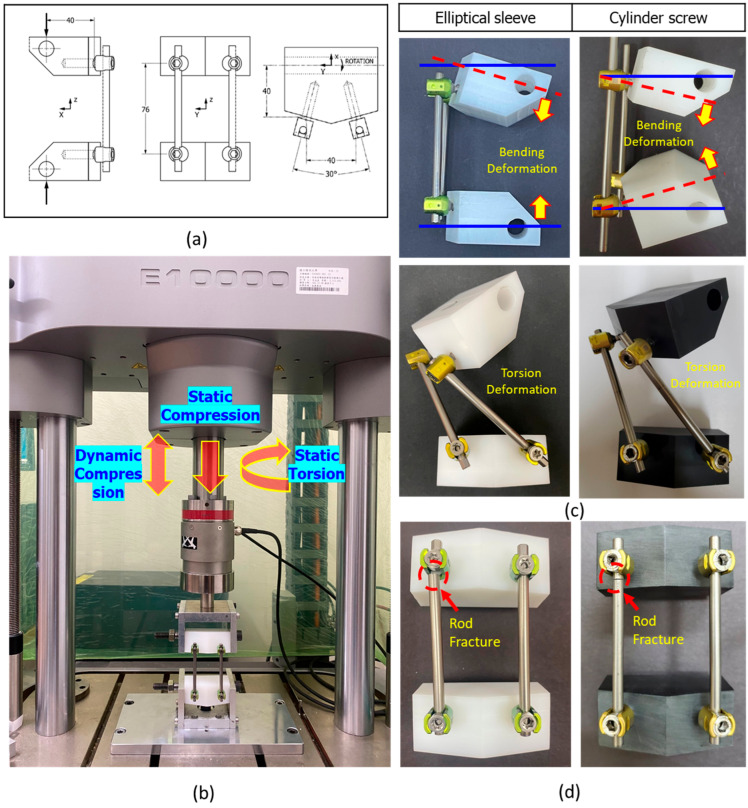
(**a**) The dimensions of the polyethylene blocks and pedicle screw implantation positions; (**b**) the actual experimental setup of ASTM F1717-21 testing; (**c**) illustrates the permanent deformation of the test samples under static compression and torsion; (**d**) fatigue test of 45% of the ultimate strength found failure occurred at the proximal rod–screw interface, leading to fractures.

The test samples were prepared in accordance with ASTM F1717 specifications. Four conventional cylindrical pedicle screws and four elliptical sleeve-integrated pedicle screws (both 4.5 mm in diameter and 35 mm in length) were each implanted into two ultra-high molecular weight polyethylene (UHMWPE) blocks (hereafter referred to as polyethylene blocks). The dimensions and material properties of the polyethylene blocks were consistent with ASTM F1717-21 (Figure 6a). The implantation process for the elliptical sleeve followed the implantation steps outlined in Section 2.3. The pedicle screws were set at an angle of 30° between the two screws. The two polyethylene blocks were then assembled using a 5.5 mm diameter, 95 mm long rod. During assembly, the upper and lower surfaces of the polyethylene blocks were aligned to ensure parallelism, and the centers of the pedicle screws on the same side were spaced 76 mm apart. Locking screws were tightened into the screw heads to secure the rod, completing the preparation of a sample (Figure 6a). The assembled test sample was further secured by inserting metal cylindrical pins into the front holes of the polyethylene blocks and connecting the upper and lower blocks to U-shaped fixtures, forming a hinge joint. The lower U-shaped fixture was fixed to the testing platform of the machine, while the upper U-shaped fixture was connected to the load cell beneath the actuator of the materials testing machine (Instron E10000, Instron, Canton, MA, USA). The actual experimental setup is shown in Figure 6b.

For static compression testing, a preload of 5 N was applied, followed by compression at a speed of 5 mm/min until the sample failed (n = 5). Failure was defined as the point at which the sample exhibited permanent deformation or slippage between components. The test recorded the maximum compressive load value (ultimate strength), yield strength, and bending stiffness. Fatigue compression testing was conducted by applying cyclic loads corresponding to 60%, 55%, 50%, 45%, and 40% of the maximum load value at which the pedicle screw system failed during static testing. The goal was to identify the load level at which the system could withstand over 5 million cycles (40% of the maximum load). Three samples were tested under these conditions to confirm that the load could sustain 5 million cycles of bending moment. The ratio of maximum to minimum load was set at R = 10 (F_max_/F_min_ = 10), with a sinusoidal waveform and a dynamic loading frequency of 5 Hz. The experimental setup for fatigue testing was identical to that of static testing. The stopping criteria for fatigue testing included sample failure, reaching 5 million cycles, or vertical downward deformation exceeding the safety displacement limit of 30 mm. A load-life curve was generated to illustrate the performance of the pedicle screw system under different loads and cycles.

For static torsion testing, a preload of 1 N-m was applied, followed by torsion at a rate of 60°/min until the sample failed. Failure was defined as the point at which the sample exhibited permanent deformation. The test recorded the maximum ultimate torsional strength, angle of failure, and other parameters.

### 2.5. Biomechanical Compression Fatigue and Pull-Out Testing

**Figure 7:**
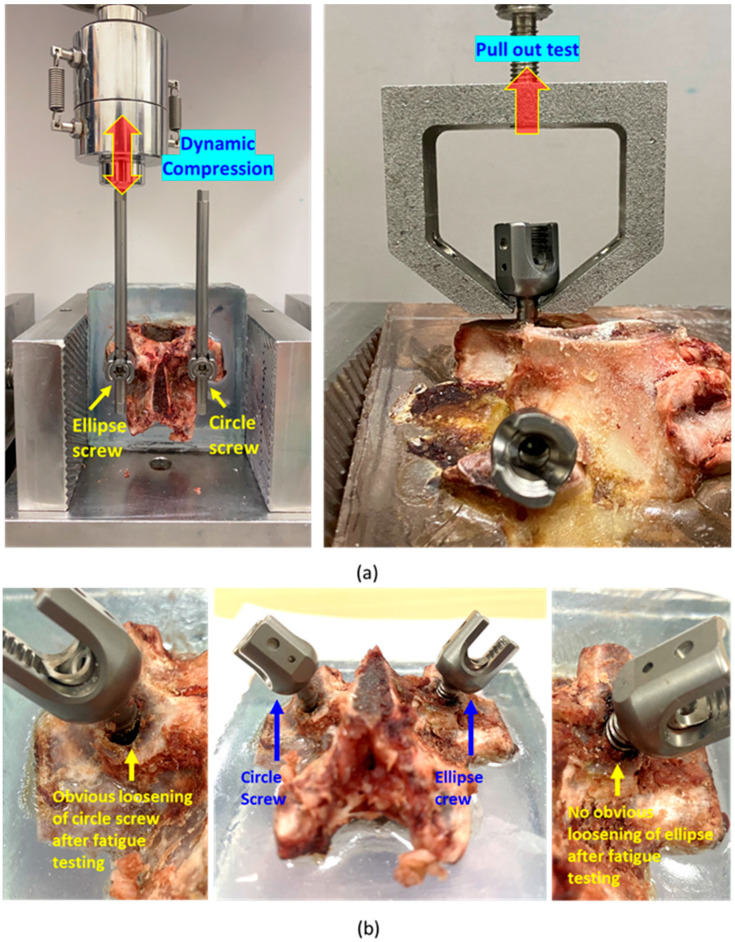
(**a**) (**Left**): illustration of biomechanical fatigue compressive test on the connection rod and (**right**): illustration of pedicle screw pull-out testing; (**b**) failure modes after compression fatigue testing, (**left**): cylinder screw, (**right**): ellipse sleeve screw.

To verify the stability and retention strength of the newly developed pedicle screw system after implantation into the vertebrae, six porcine vertebrae (L2, L3, L4) were randomly selected as experimental specimens. These vertebrae were chosen because their cross-sectional shape was elliptical [25], aligning with the study’s research objectives. Subsequently, a conventional cylindrical pedicle screw and the newly developed pedicle screw system (with the elliptical sleeve) were implanted on the right and sides of each vertebra, respectively (Figure 7a). The anterior portion of each vertebra was embedded in epoxy resin, and a 5.5 mm diameter rod was secured to the heads of the pedicle screws on both sides. The experimental specimens were fixed on a materials testing machine (Instron E3000, Instron, Canton, MA, USA), with the embedded resin block clamped using a vise, and a load applicator connected to a load cell was aligned to apply force to the proximal end of the rod. Fatigue loading was applied at 3 Hz with a force range of 40 N to 400 N.

The six specimens were randomly divided into two groups (n = 3). In the first group, the rods attached to the cylindrical pedicle screw and the pedicle screw system with the elliptical sleeve were subjected to 5000 fatigue loading cycles. In the second group, the same procedure was performed for 100,000 fatigue cycles.

After the fatigue loading tests, each vertebral specimen was rotated 90 degrees and re-secured on the testing machine. A custom load applicator connected to the load cell was used to clamp the screw head. A pull-out test of the pedicle screws was performed at a speed of 3 mm/min, and the maximum pull-out force and force-displacement curves were recorded (Figure 7a). For the specimens that underwent 100,000 fatigue cycles, the posterior portion of the vertebrae was subsequently embedded in epoxy resin. The resin-embedded block was carefully trimmed using a low-speed diamond blade saw, preserving only the pedicle and the adjacent portion of the vertebral body containing the screw. To enhance visualization of the interfacial gap, the trimmed sample underwent a second embedding process using epoxy resin mixed with a small amount of commercial blue dye. During this process, the dyed resin naturally flowed into any existing gaps between the screw and bone, thereby enabling the visualization of even very small interfacial gaps. Subsequently, the fully embedded sample was sectioned again axially; this cutting plane was precisely controlled to expose the bone–screw interface while preserving the original structural relationships. This entire procedure was intended as a qualitative demonstration to visualize the gap, rather than a quantitative analysis.

## 3. Results

### 3.1. FE Analysis

After applying a 100 N vertical compressive load, the maximum principal stress in the pedicle cross-section was analyzed. The pedicle screw with an elliptical sleeve exhibited a maximum stress of 8.26 MPa, whereas the cylindrical screw reached 9.97 MPa, indicating that the stress at the pedicle–screw interface was approximately 1.21 times higher with the cylindrical screw (Figure 2b). Under a 1 N·m torque load, the elliptical sleeve screw recorded a maximum stress of 1.56 MPa, while the cylindrical screw exhibited 2.30 MPa, meaning the stress was approximately 1.9 times higher in the cylindrical design (Figure 2b). Furthermore, the elliptical sleeve screw demonstrated a more uniform stress distribution, effectively reducing localized stress accumulation.

### 3.2. ASTM F1717 Testing

The static compression test results indicated that the elliptical sleeve pedicle screw, compared to the cylindrical screw, exhibited higher values in ultimate strength, yield strength, and bending stiffness (Table 2). This suggests that the elliptical sleeve screw has greater resistance to bending loads, allowing it to withstand larger deformations without experiencing material yielding or failure. Although in the static torsion test, the torsional ultimate strength and yield strength of the elliptical sleeve pedicle screw were lower than those of the cylindrical screw, its torsional stiffness remained higher. This implies that the elliptical sleeve screw provides better resistance to rotational forces per unit of angular displacement. Figure 6c illustrates the permanent deformation of the test samples under static compression and torsion, showing no fractures in either the screws or the connection rods.

**Figure 8:**
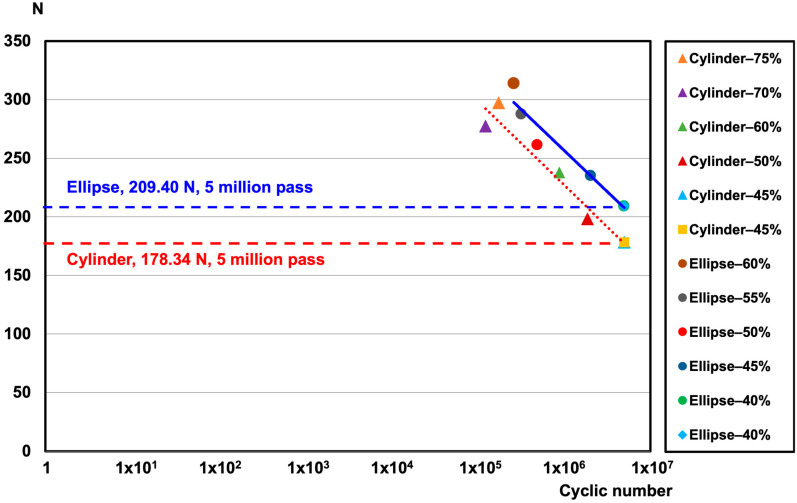
Fatigue S-N curve of ASTM F1717 testing for pedicle cylinder and ellipse sleeve screws.

In the dynamic compression fatigue test, both the elliptical sleeve and cylindrical pedicle screws successfully withstood 5 million load cycles at 40% of their ultimate strength, which corresponded to 209.40 N for the elliptical sleeve screw and 178.34 N for the cylindrical screw, without failure (Figure 8). The fatigue test further demonstrated that the elliptical sleeve screw had superior resistance to bending moments. Conversely, at 45% of the ultimate strength (235.54 N for the elliptical sleeve screw and 175.46 N for the cylindrical screw), failures occurred at the proximal rod–screw interface, leading to fractures (Figure 6d).

### 3.3. Biomechanical Pull-Out Testing

**Figure 9:**
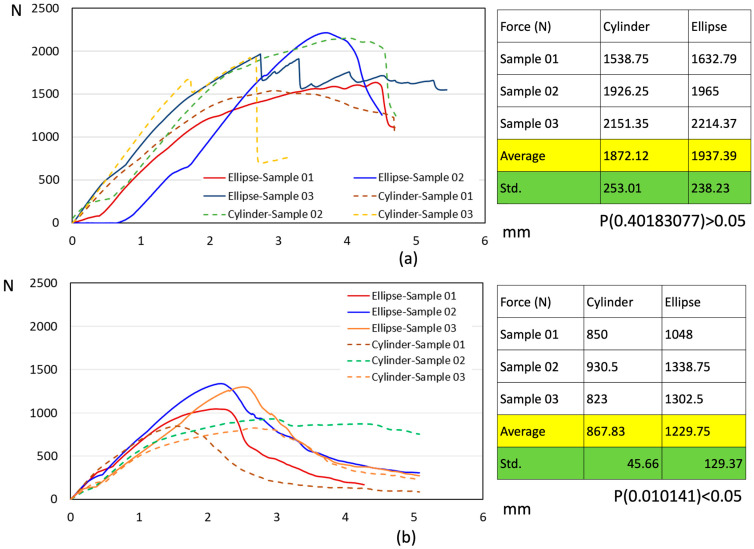
(**a**) Force and displacement and corresponding values of static pull-out testing after 5000 fatigue compression testing; (**b**) force and displacement and corresponding values of static pull-out testing after 100,000 fatigue compression testing.

This study compared the pull-out strength of elliptical sleeve pedicle screws and traditional cylindrical pedicle screws after undergoing different cycles of 200 N–400 N dynamic compressive fatigue testing, evaluating their fixation stability and durability under dynamic loading conditions. The results indicated that after a short-term fatigue cycle of 5000 repetitions, the average pull-out strength of the elliptical sleeve screw and cylindrical screw was 1937.39 N and 1872.12 N, respectively (Figure 9a). Although the pull-out strength of the elliptical sleeve screw was slightly higher, statistical analysis showed *p* = 0.4018 (>0.05), indicating no significant statistical difference between the two groups. However, as the fatigue cycle increased to 100,000 repetitions, a significant difference emerged. The average pull-out strength of the elliptical sleeve screw remained at 1229.75 N, whereas the cylindrical screw decreased to 867.83 N (Figure 9b). Statistical analysis revealed *p* = 0.0101 (<0.05), demonstrating a significantly superior fixation strength of the elliptical sleeve screw compared to the cylindrical screw. These findings highlight the superior retention strength of the elliptical sleeve screw, suggesting its enhanced fixation performance in long-term stress environments compared to conventional cylindrical screws.

**Figure 10:**
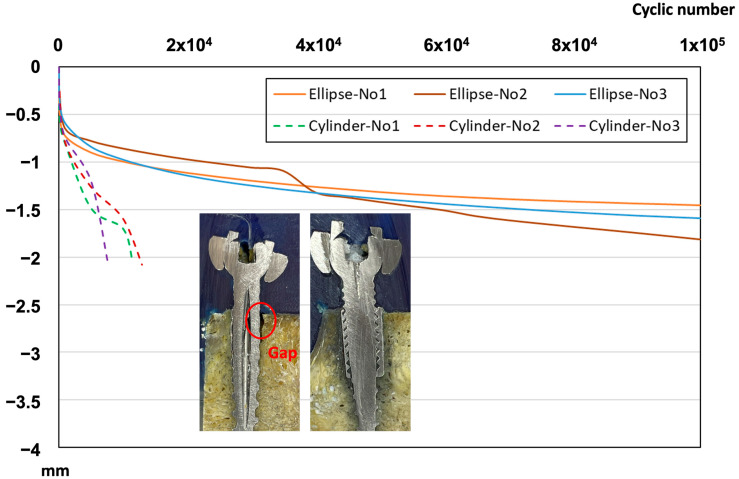
Fatigue displacement–cyclic load number curve of biomechanical fatigue curves for both pedicle cylinder and ellipse sleeve screws and one of corresponding on the behalf of failure modes.

The results of long-term fatigue displacement behavior of elliptical sleeve pedicle screws and traditional cylindrical pedicle screws under dynamic compressive loading conditions showed that cylindrical screw reached the preset displacement threshold of 2 mm after an average of 10,663 loading cycles, leading to test termination. In contrast, the elliptical sleeve screw maintained stable displacement levels and successfully withstood over 100,000 loading cycles, demonstrating superior durability under prolonged loading conditions (Figure 10). The failure model of two pedicle screws and their corresponding cross-sectional imaging further revealed key differences between the two screw designs (Figure 7b). In the cylindrical screw, a noticeable gap formed at the bone–screw interface, indicating that the screw gradually loosened under prolonged fatigue loading, leading to fixation instability. Conversely, the elliptical sleeve screw (right image) exhibited a tight bone–screw interface with no apparent gaps, suggesting superior bone integration and enhanced fixation stability over time (Figure 10).

## 4. Discussion

Pedicle screws are one of the most essential internal fixation devices in spinal stabilization surgery [1,2,3,4]. To improve clinical success rates, current modifications of pedicle screws primarily focus on enhancing the fixation mechanism, altering thread design, and increasing screw length and diameter [18,19,20,21,22]. For example, fixation strength can be improved by incorporating expansion mechanisms at the screw’s distal end or combining bone cement augmentation [18]. Additionally, changes in thread types were designed to increase the bone–screw contact area and enhanced holding strength. However, patients with low bone density may not achieve sufficient fixation strength, and increasing the screw dimensions is often constrained by vertebral anatomical structures, limiting its applicability. Furthermore, while optimizing thread design helps distribute stress more effectively, inadequate bone quality may still lead to long-term micromotion, ultimately resulting in screw loosening or breakage.

The cross-sectional morphology of the pedicle in the thoracolumbar spine is elliptical, and literature has highlighted the critical role of the pedicle in screw stability [14,15,16]. However, to date, no studies have proposed designing pedicle screws with an elliptical shape that closely matches the pedicle’s cross-section. This is likely due to challenges associated with screw implantation and the necessary instrumentation. This study introduces a modular elliptical sleeve that can be assembled onto a traditional cylindrical screw. By utilizing specially designed instruments, both the screw and the elliptical sleeve can be effectively implanted into the vertebral body and pedicle, enhancing geometric compatibility with the bone and improving fixation strength at the pedicle region.

From the perspective of material mechanics [17], when a bone screw is implanted into bone—a composite material—the bending moment and torque applied to its cross-section result in bending stress and shear stress, respectively. These stresses are inversely proportional to the fourth power of the second moment of inertia (Figure 1). This means that as the second moment of inertia increases, both bending stress and shear stress decrease, improving the screw’s structural resistance to deformation. Assuming that the radius of a cylindrical pedicle screw and the minor axis of the elliptical sleeve are both a, and the major axis of the elliptical sleeve is 1.2a, the second moment of inertia under bending moment for the cylindrical and elliptical configurations are 0.785a^4^ and 1.357a^4^, respectively. This indicates that the elliptical configuration provides approximately 1.44 (1/0.694 in Figure 1) times the bending strength of the cylindrical design. Under torque, the second moment of inertia for the cylindrical and elliptical configurations are 0.785a^4^ and 2.29848a^4^, respectively, suggesting that the elliptical design has approximately 2.44 T(1/0.409 in Figure 1) times the torsional strength of the cylindrical screw. Therefore, theoretically, incorporating an elliptical sleeve onto the pedicle screw at the pedicle region allows it to endure greater stress compared to a cylindrical screw, reducing the load transferred to the surrounding bone and potentially enhancing screw stability.

The FE model used in this study is a simplified representation of spinal anatomy and does not incorporate full validation against cadaveric or in vivo biomechanical data. However, the purpose of including the FE analysis was not to derive absolute or clinically predictive values, but rather to evaluate the relative mechanical behavior of our novel screw compared to the conventional design. The model was intended to highlight qualitative trends in stress distribution and deformation, which were used to guide the mechanical design and prototyping phase of screw development. Importantly, the FE analysis served as a preliminary design verification tool to justify further physical testing. FE analysis revealed a similar trend to the theoretical calculations. However, under bending moment and torque, the increase in stress resistance for the cylindrical screw was lower than the theoretically predicted values. While the theoretical calculations suggested improvements of 1.44 times for bending and 2.44 times for torque, the FE analysis results showed 1.21 times and 1.91 times, respectively. This discrepancy arises from the complexity of the vertebral geometry in the FEA model, which is not as simplified as the idealized geometric assumptions used in theoretical calculations. Nevertheless, these results support the theoretical foundation for the elliptical sleeve design, highlighting its potential advantages in pedicle screw applications. The elliptical sleeve design effectively leverages the biomechanical benefits of an elliptical cross-section, optimizing stress distribution and reducing stress concentration at the bone–screw interface. This, in turn, may help mitigate the risk of screw loosening or failure, improving long-term fixation stability.

The ASTM F1717 test is primarily used to evaluate the static strength, bending stiffness, and fatigue resistance of spinal implants under loading conditions. This testing standard is also an important criterion for FDA (U.S. Food and Drug Administration) pre-market evaluation of spinal fixation devices, ensuring that implant designs meet internationally recognized performance benchmarks. However, it is important to note that the actual in vivo loads experienced by implants may differ from the controlled conditions in standardized mechanical tests [24]. These test methods simplify the complex biomechanical environment within the body, which involves factors such as bone integration, micromotion, and bone remodeling. As a result, ASTM F1717 test outcomes cannot directly predict the long-term performance of implants in clinical applications, but they do provide a valid comparative basis for different screw designs, allowing us to assess the biomechanical advantages of the elliptical sleeve pedicle screw compared to traditional cylindrical screws.

The test results demonstrated that the elliptical sleeve screw exhibited superior static compressive and torsional stiffness compared to the cylindrical screw. Additionally, in fatigue compression testing, the elliptical sleeve screw withstood a maximum load of approximately 235.4 N over 5 million cycles, which was significantly higher than the 175.46 N observed for the cylindrical screw. These findings confirm that the elliptical sleeve design meets FDA substantial equivalence testing requirements, further supporting its potential as an improved alternative for pedicle screw fixation.

In the biomechanical fatigue testing, the displacement curves revealed that cylindrical screws exhibited a large slope of displacement increase, indicating that micro-motion at the bone–screw interface likely began at an early stage. This micro-motion contributed to bone structure degradation, ultimately compromising fixation stability. On average, cylindrical screws reached the 2 mm displacement threshold after 10,663 cycles, at which point they were considered failed due to accumulated micro-motion leading to bone interface breakdown. In contrast, all three samples of the elliptical sleeve screws successfully completed 100,000 cycles, with relatively stable displacement behavior. The anatomical compatibility and improved stress distribution of the elliptical sleeve design contributed to lower displacement variations, significantly enhancing long-term durability and fixation stability. These findings further support the elliptical sleeve screw as an improved design, demonstrating clear advantages in reducing screw micro-motion and minimizing bone interface degradation, which may offer greater potential in clinical applications.

Additionally, this study utilized porcine spines, particularly in the thoracolumbar region (T12-L5) [25], where elliptical cross-sectional characteristics closely resemble those of the human lumbar spine. This choice provided a more accurate biomechanical evaluation, as the bone density and mechanical properties of porcine spines are also comparable to human vertebrae, making them a commonly used animal model in biomechanical research. The 100,000-cycle fatigue testing in this study approximates two weeks to one month of physiological spinal motion [24,26], which, while not fully replicating long-term clinical conditions, provides an early indication of fixation stability trends. Future studies should extend longer-term dynamic fatigue testing to further investigate changes in the bone–screw interface over time. Notably, the 2 mm displacement threshold has been widely adopted in the literature as a criterion for premature screw failure [27,28], further emphasizing the superior performance of the elliptical sleeve screw in preventing early fixation loss.

Traditional cylindrical pedicle screws have long been the standard in spinal fixation, with well-established implantation instruments that allow for straightforward placement through the use of guidewires, pre-drilled pilot holes, and screw-insertion techniques. However, while an elliptical cross-section screw is more anatomically compatible with the pedicle morphology, it cannot solely rely on existing screw-insertion techniques to achieve stable fixation. To ensure feasibility and safety during surgical implantation, this study developed a dedicated implantation instrument specifically designed for the elliptical sleeve screw. To overcome the challenges associated with elliptical sleeve screw placement, we developed a customized bone-shaping instrument. This device allows for the precise preparation of an elliptical pedicle pathway while preserving the structural integrity of the pedicle. Once the bone tunnel is shaped, the screw body is inserted in a conventional manner, ensuring that the elliptical sleeve aligns and engages with the pedicle. By employing a combination of gradual threading and a press-fit mechanism, the elliptical sleeve is progressively embedded into the bone without rotational misalignment. Previous biomechanical studies [20] have shown that the screw thread in the pedicle region contributes less significance in fixation strength than previously assumed. Press-fit mechanism demonstrated improved mechanical performance in terms of holding power and micromotion resistance. These findings supported our decision to prioritize press-fit fixation via the elliptical sleeve over traditional thread engagement in the pedicle region. In our system, the fine thread functions as an interlocking mechanism to securely carry and engage the modular elliptical sleeve during insertion into the pre-shaped elliptical tunnel. The fine thread ensures that the sleeve is delivered with proper orientation and alignment and prevents premature disengagement or misplacement during implantation. Once seated, the sleeve provides a press–fit interface with the pedicle wall, which serves as the primary fixation mechanism at the pedicle region. Further evaluation of its clinical usability and surgeon adaptation will be necessary to optimize its integration into routine spinal procedures.

This study primarily evaluated the biomechanical performance of the innovative elliptical sleeve pedicle screw but did not analyze its biological interactions with bone, such as osseointegration or bone remodeling. Future investigations should incorporate histological studies or micro-computed tomography (Micro-CT) analysis to determine whether this design can promote bone growth and enhance long-term stability at the bone–screw interface. Currently, pedicle screws are predominantly made of titanium alloy, but the use of different surface coatings or bioactive materials (e.g., hydroxyapatite coatings) could further improve osseointegration and fixation strength. Future research should explore optimized material selections to enhance clinical outcomes. Additionally, long-term in vivo animal studies are required to assess bone integration, screw loosening rates, and potential effects on surrounding tissues. These studies will also help evaluate postoperative complications and long-term stability, which are critical before transitioning to clinical trials. Further investigations should explore the clinical applicability of the elliptical sleeve screw in various surgical indications, including scoliosis correction, traumatic fracture fixation, and tumor reconstruction surgeries. Expanding its clinical indications through comprehensive testing may provide a novel alternative for improving spinal fixation stability.

## 5. Conclusions

This study introduced a novel modular pedicle screw system incorporating an elliptical sleeve to enhance fixation strength at the pedicle. By leveraging the biomechanical advantages of an elliptical cross-section, FE analysis confirmed that the elliptical sleeve significantly increased resistance to bending moments and torsional loads. ASTM F1717 testing further validated the superior static and fatigue performance of the elliptical sleeve screw, particularly in maintaining fixation under cyclic loading conditions. Biomechanical testing using porcine spines revealed that the elliptical sleeve screw exhibited significantly lower micromotion and higher pull-out strength after long-term cyclic loading, suggesting its potential to improve implant longevity and reduce the risk of screw loosening. While this study primarily focused on the mechanical performance of the new screw design, these findings highlight the potential of the elliptical sleeve pedicle screw as an innovative alternative for spinal fixation.

## Figures and Tables

**Table 1:** Materials properties used in FE analysis.

Material	Young’s Modulus (MPa)	Poission’s Ratio
Cortical bone	12,000	0.3
Cancellous	100	0.2
Endplate	24	0.25
Ti6Al4V	110,000	0.3

**Table 2:** Results of ASTM F1717 for static compression and torsion testing.

Testing Type	Static Compression						Static Torsion					
Recorded	Ultimate Strength (N)		Yield Strength (N)		Bending Stiffness (N/mm)		Ultimate Torque (N-m)		Yield Torque (N-m)		Torsional Stiffness (N-m/Degree)	
Sample type	Ellipse	Cylinder	Ellipse	Cylinder	Ellipse	Cylinder	Ellipse	Cylinder	Ellipse	Cylinder	Ellipse	Cylinder
Sample 01	455.82	357.20	385.42	267.90	34.97	22.14	13.82	17.05	8.82	13.8	2.81	2.14
Sample 02	556.40	365.70	406.89	333.30	31.20	26.36	15.49	18.99	9.30	14.04	2.56	2.00
Sample 03	528.68	460.90	404.51	299.90	30.72	27.82	13.81	16.38	9.79	12.75	2.32	2.07
Sample 04	558.68	407.90	422.16	252.10	32.31	24.85	13.80	17.14	9.25	12.96	2.41	2.13
Sample 05	517.60	389.90	360.42	217.90	30.24	27.19	16.32	17.77	10.45	13.4	2.36	2.07
Average (Std.)	523.43 (41.72)	357.20 (41.27)	395.88 (23.73)	267.90 (44.30)	31.89 (1.89)	22.14 (2.27)	14.23 (0.84)	17.47 (0.98)	9.29 (0.40)	13.39 (0.54)	2.41 (0.12)	2.08 (0.06)

## Data Availability

The data presented in this study are available on request from the corresponding author.
